# Association between weekend catch-up sleep and gallstone disease in US adults: a cross-sectional study from NHANES 2017–2020

**DOI:** 10.3389/fpubh.2025.1573858

**Published:** 2025-05-19

**Authors:** Jianwei Cao, Weishuai Zhang, Jixuan Yi, Yang Zhang, Xiaoyan Tong, Xiangnan Zhu

**Affiliations:** Department of General Surgery, Fourth Affiliated Hospital of Nanchang University, Nanchang, China

**Keywords:** NHANES, weekend catch-up sleep, sleep, gallstones, smoking

## Abstract

**Background:**

Gallstones are the most prevalent cause of hospitalization among digestive disorders. For humans, sleep is an essential physiological function. The relationship between gallstones and sleep is well established, but the consequences of weekend catch-up sleep (WCS) on gallstones remain unclear. This research examined the connection between gallstone disease and WCS.

**Methods:**

We included 6,957 participants from the 2017–2020 National Health and Nutrition Examination Survey (NHANES) who met the eligibility criteria and had complete data. Logistic regression, restricted cubic spline, and subgroup analyses were employed to assess the relationship between the presence of gallstones and WCS.

**Results:**

Our study indicated that trouble sleeping and late sleep were risk factors for gallstones in a model adjusted for all covariates. The restricted cubic spline results revealed that WCS was negatively correlated linearly with gallstone disease. Additionally, subgroup analysis showed a statistically significant relationship between WCS > 2 hours and a lower risk of gallstones, particularly among non-smokers and males.

**Conclusion:**

Our study results demonstrate that trouble sleeping and late sleep increase the incidence of gallstones. In addition, the protective effect of WCS > 2 hours on reducing the incidence of gallstones is particularly significant in individuals who are non-smokers and males, whereas in the smoking population, WCS < 0 hours serves as a protective factor against gallstones.

## 1 Introduction

Gallstones have become a common digestive disorder among adults in the United States, with an incidence rate of 10–15% (1). Although nearly 80% of gallstone patients remain asymptomatic, the estimated risk of complications in asymptomatic patients is approximately 0.1–0.3% annually, whereas in individuals who have experienced their first episode of colic, the annual risk increases to between 1 and 3% (2, 3). Once symptoms occur, surgery is the primary treatment for gallstones (4). Prior research has demonstrated a significant relationship between gallstones and factors such as being female, race, increased body mass index (BMI), obesity, and vitamin C deficiency (5–8).

Sleep is a crucial physiological process that influences various metabolic and endocrine functions (9), including hormone control, cardiovascular health, energy conservation, glucose management, muscle regeneration, tissue growth, protein synthesis, and cognitive function (10–12). According to the international categorization of sleep disorders, 7–8 h is the ideal amount of sleep duration (13). Previous studies have shown that insufficient or excessive sleep duration is associated with lower bone mineral density, osteoporosis, and a higher prevalence of depression (14–17). The disparity between the duration of sleep on weekends and weekdays is referred to as weekend catch-up sleep (WCS) (18). WCS is significantly linked to obesity prevention (19), decreased incidence of cardiovascular disease (20), lower risk of hypertension (21), reduced odds of depressive symptoms (22), and reduced levels of high-sensitivity C-reactive protein (23).

A recent study has shown a close relationship between sleep and gallstones (24), but studies on the relationship between gallstones and WCS remain lacking. By examining data from the National Health and Nutrition Examination Survey (NHANES) from 2017 to March 2020, we aimed to explore the correlation between gallstones and WCS and provide new insights into the prevention of gallstone disease.

## 2 Methods

### 2.1 Research population

**Figure 1:**
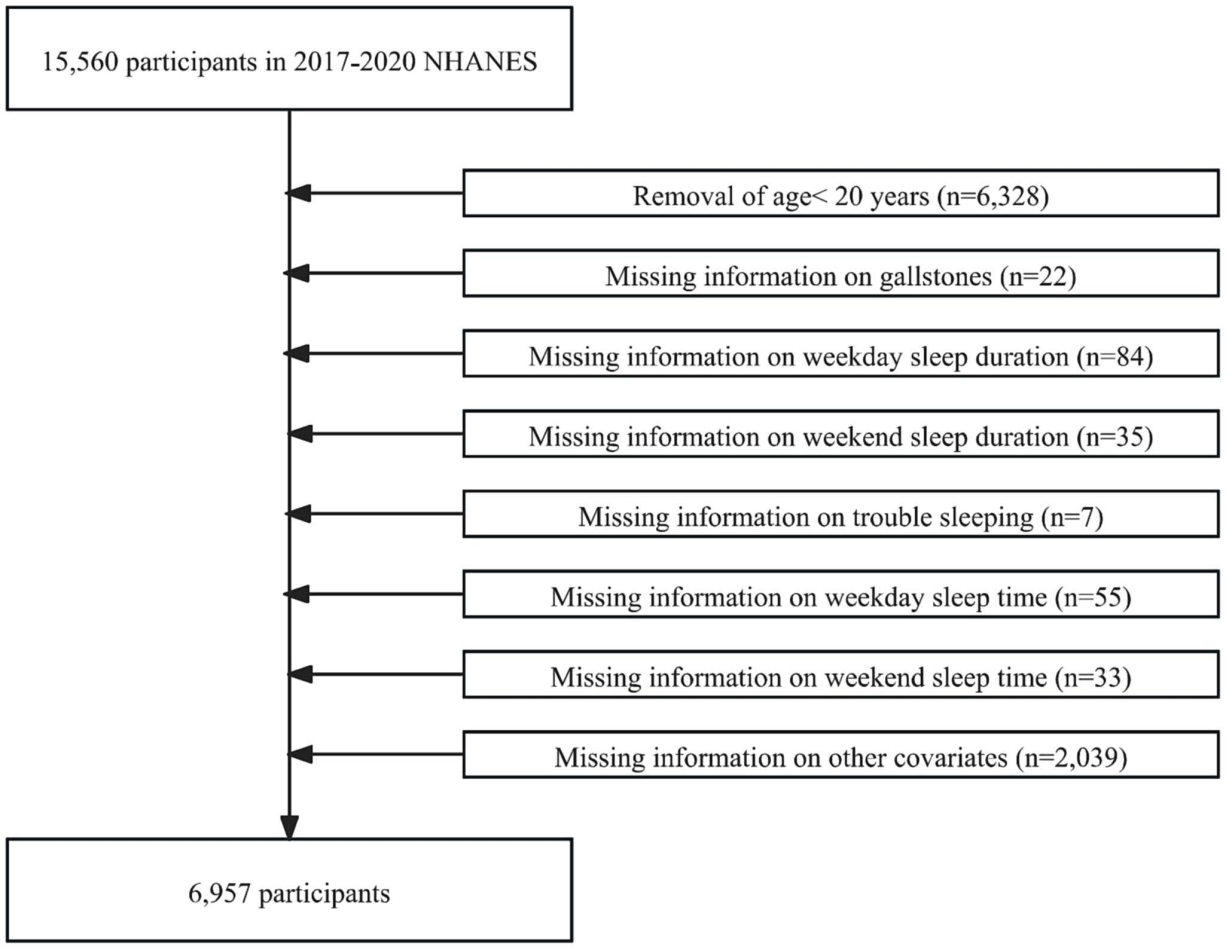
Flowchart of participant selection.

The NHANES is a representative health survey of the United States. Its comprehensive data make it a key resource for studying chronic disease, nutrition, and population health. Our study analyzed data from the 2017–2020 NHANES survey participants. In the beginning, 15,560 participants were enrolled in our study. However, we excluded participants younger than 20 years of age (*n* = 6,328), those with missing information about gallstones (*n* = 22), weekday sleep duration (*n* = 84), weekend sleep duration (*n* = 35), trouble sleeping (*n* = 7), weekday sleep time (*n* = 55), weekend sleep time (*n* = 33), education level (*n* = 13), BMI (*n* = 811), diabetes (*n* = 3), hypertension (*n* = 12), smoking (*n* = 4), and alcohol consumption (*n* = 1,196). Ultimately, 6,957 participants with no missing information were included in this study (Figure 1).

### 2.2 Defining gallstone disease

We used the question “Has a doctor or other health professional ever told you that you had gallstones?” to determine whether participants had gallstones. If the participant answered “Yes,” it meant the presence of gallstones; if “No,” it meant the absence of gallstones.

### 2.3 Definition of WCS and other sleep factors

Weekday and weekend sleep durations were obtained from the questions on “Number of hours usually slept on weekdays” and “Number of hours usually slept on weekends,” respectively. We categorized both weekday sleep duration and weekend sleep duration into three groups: normal (<7 h per night), short (7–9 h per night), and long (>9 h per night) (25). WCS was defined as the difference between weekend sleep duration and weekday sleep duration. We categorize WCS into four groups: <0 h, =0 h, 0–2 h, and >2 h (20). Weekday sleep time and weekend sleep time were obtained from the questions “What time do you usually fall asleep on weekends?” and “What time do you usually fall asleep on weekends?.” We categorized both weekday sleep duration and weekend sleep duration into three groups: normal sleep time (20:00–23:00), late sleep (>23:00), and abnormal sleep time (>03:00). Trouble sleeping was determined by the question “Have you ever told a doctor or other health professional that you have trouble sleeping?.” If the participant answered “Yes,” it meant the presence of trouble sleeping; if “No,” it meant the absence of trouble sleeping.

### 2.4 Identification of covariates

In our study, the following variables were employed as covariates: gender, age, BMI, race, alcohol consumption, education level, smoking status, hypertension, and diabetes. We divided the ages into four groups: <35 years, 35–49 years, 50–64 years, and ≥65 years. We categorized the races into five groups: Mexican American, Non-Hispanic White, Other Hispanic, Non-Hispanic Black, and Other Race. We categorized education levels into three groups: <High school (below the 12th grade and those who did not obtain a high school diploma), High school [high school graduate/general educational development (GED) or equivalent], and >High school (some college or associate’s degree, college graduate or above). BMI was stratified into two groups: <30 kg/m^2^ and ≥30 kg/m^2^. The questions “Doctor told you had hypertension” and “Doctor told you had diabetes” were used to determine whether a participant had hypertension or diabetes. Smoking status was determined by the question “Have you smoked at least 100 cigarettes in your entire life?.” The responses “Yes” and “No” identified smokers and non-smokers, respectively. Alcohol consumption status was determined based on the question “Ever drink 4/5 cups or more a day?.” The responses “Yes” and “No” identified drinkers and non-drinkers, respectively.

### 2.5 Statistical analyses

Categorical variables were presented as numbers (*n*) and percentages (%). For categorical variables, the chi-square test was used to compare the characteristics between participants with and without gallstones. Our study utilized logistic regression to calculate the odds ratios (OR) and corresponding 95% confidence intervals (CI) for the association between WCS, other sleep factors, and gallstones. Three logistic regression models were constructed to further explore the potential links between them. Model 1 was unadjusted for covariates. Model 2 race, age, and gender were adjusted. Model 3 race, age, gender, BMI, education level, diabetes, hypertension, smoking, and alcohol consumption status were adjusted. Additionally, to find any potential non-linear dose–response relationship between WCS and gallstone disease, we employed a restricted cubic spline (RCS) model. Covariates, namely gender, race, education level, BMI, diabetes, smoking, and alcohol consumption, were adjusted when constructing this model. Finally, we performed subgroup analyses to examine the association between WCS and gallstones in the different groups. The R program (4.4.2) was utilized for our study, and *p* < 0.05 was defined as statistically significant.

## 3 Results

### 3.1 Baseline characteristics of participants

The characteristics of the variables involved in this study are summarized in Table 1. After applying our exclusion criteria, there were 6,957 participants in the study, including 728 participants with gallstones and 6,229 participants without gallstones. The participants with gallstones tended to be female (*p* < 0.001), older (*p* < 0.001), Non-Hispanic White (*p* < 0.001), have a BMI ≥ 30 kg/m^2^ (*p* < 0.001), smokers (*p* = 0.005), have trouble sleeping (*p* < 0.001), WCS = 0 (*p* < 0.001), sleep time >23:00 on weekdays (*p* = 0.002), have diabetes (*p* < 0.001) and hypertension (*p* < 0.001) compared to participants without gallstones.

**Table 1:** Baseline characteristics of participants.

Characteristic	Non-gallstone (*n* = 6,229)	Gallstone (*n* = 728)	*p*-value
Gender			<0.001
Male	3,324 (53.4%)	218 (29.9%)	
Female	2,905 (46.6%)	510 (70.1%)	
Age, years			<0.001
<35	1,569 (25.2%)	65 (8.9%)	
35–49	1,493 (24%)	152 (20.9%)	
50–64	1748 (28.1%)	231 (31.7%)	
≥65	1,419 (22.8%)	280 (38.5%)	
Race			<0.001
Mexican American	731 (11.7%)	95 (13%)	
Other Hispanic	623 (10%)	87 (12%)	
Non-Hispanic White	2,265 (36.4%)	326 (44.8%)	
Non-Hispanic Black	1,692 (27.2%)	144 (19.8%)	
Other Race	918 (14.7%)	76 (10.4%)	
Education level			0.631
High school	1,497 (24%)	186 (25.5%)	
<High school	1,019 (16.4%)	120 (16.5%)	
>High school	3,713 (59.6%)	422 (58%)	
BMI, kg/m^2^			<0.001
<30	3,638 (58.4%)	272 (37.4%)	
≥30	2,591 (41.6%)	456 (62.6%)	
Diabetes			<0.001
No	5,380 (86.4%)	540 (74.2%)	
Yes	849 (13.6%)	188 (25.8%)	
Hypertension			<0.001
No	3,955 (63.5%)	332 (45.6%)	
Yes	2,274 (36.5%)	396 (54.4%)	
Alcohol consumption			0.606
No	5,272 (84.6%)	622 (85.4%)	
Yes	957 (15.4%)	106 (14.6%)	
Smoking			0.005
No	3,446 (55.3%)	362 (49.7%)	
Yes	2,783 (44.7%)	366 (50.3%)	
Sleep duration on weekdays, hours			0.687
7–9	3,958 (63.5%)	459 (63%)	
<7	1,633 (26.2%)	187 (25.7%)	
>9	638 (10.2%)	82 (11.3%)	
Sleep duration on weekends, hours			0.148
7–9	3,814 (61.2%)	466 (64%)	
<7	993 (15.9%)	119 (16.3%)	
>9	1,422 (22.8%)	143 (19.6%)	
WCS, hours			<0.001
0	2,326 (37.3%)	328 (45.1%)	
<0	1,009 (16.2%)	103 (14.1%)	
0–2	2012 (32.3%)	228 (31.3%)	
>2	882 (14.2%)	69 (9.5%)	
Trouble sleeping			<0.001
No	4,480 (71.9%)	406 (55.8%)	
Yes	1749 (28.1%)	322 (44.2%)	
Sleep time on weekdays			0.002
20:00–23:00	4,067 (65.3%)	455 (62.5%)	
>23:00	1826 (29.3%)	250 (34.3%)	
>03:00	336 (5.4%)	23 (3.2%)	
Sleep time on weekends			0.824
20:00–23:00	3,238 (52%)	371 (51%)	
>23:00	2,763 (44.4%)	328 (45.1%)	
>03:00	228 (3.7%)	29 (4%)	

Categorical variables: numbers are used to express values (percentages).

BMI, body mass index; WCS, weekend catch-up sleep.

### 3.2 Association between gallstones and WCS and other sleep factors

Table 2 shows the relationship between gallstones and WCS and other sleep factors. In Model 1, when comparing with the reference group of WCS = 0 h, WCS < 0 h (OR = 0.72; 95% CI: 0.57–0.91), WCS 0–2 h (OR = 0.80; 95% CI: 0.67–0.96), and WCS > 2 h (OR = 0.55; 95% CI: 0.42–0.73) were identified as protective factors for gallstones. In Model 2, gender, age, and race were adjusted. When comparing participants with sleep time from 20:00 to 23:00 as the reference group, sleep time > 23:00 on weekdays showed 1.41 times higher risk of gallstones, as well as sleep time > 23:00 and > 03:00 on weekends, showed 1.42 times and 1.68 times higher risk of gallstones, respectively. In Model 3, all covariates were adjusted; participants with trouble sleeping showed a 1.45 times higher risk of gallstones. Furthermore, participants with sleep time > 23:00 on both weekdays (OR = 1.30; 95% CI: 1.10–1.55) and weekends (OR = 1.34; 95% CI: 1.13–1.58) exhibited a higher probability of developing gallstone disease.

**Table 2:** Association between WCS, other sleep factors, and gallstones.

Characteristic	Model 1	Model 2	Model 3
	OR (95%CI, *p*-value)	OR (95%CI, *P*-value)	OR (95%CI, *P*-value)
Sleep duration on weekdays, hours			
7–9	ref.	ref.	ref.
<7	0.99 (0.83–1.18, *p* = 0.890)	0.87 (0.72–1.05, *p* = 0.149)	1.06 (0.88–1.29, *p* = 0.529)
>9	1.11 (0.86–1.42, *p* = 0.419)	1.01 (0.78–1.30, *p* = 0.952)	0.94 (0.72–1.22, *p* = 0.621)
Sleep duration on weekends, hours			
7–9	ref.	ref.	ref.
<7	0.98 (0.79–1.21, *p* = 0.859)	1.06 (0.85–1.33, *p* = 0.579)	0.97 (0.77–1.22, *p* = 0.795)
>9	0.82 (0.68–1.00, *p* = 0.053)	0.84 (0.68–1.03, *p* = 0.092)	0.82 (0.66–1.01, *p* = 0.058)
WCS, hours			
0	ref.	ref.	ref.
<0	0.72 (0.57–0.91, *p* = 0.007)	0.97 (0.76–1.23, *p* = 0.783)	0.94 (0.73–1.20, *p* = 0.616)
0–2	0.80 (0.67–0.96, *p* = 0.017)	1.04 (0.85–1.26, *p* = 0.720)	1.05 (0.86–1.28, *p* = 0.614)
>2	0.55 (0.42–0.73, *p* < 0.001)	0.79 (0.59–1.05, *p* = 0.108)	0.78 (0.58–1.04, *p* = 0.091)
Trouble sleeping			
No	ref.	ref.	ref.
Yes	2.03 (1.74–2.38, *p* < 0.001)	1.70 (1.45–2.00, *p* < 0.001)	1.45 (1.22–1.71, *p* < 0.001)
Sleep time on weekdays			
20:00–23:00	ref.	ref.	ref.
>23:00	1.22 (1.04–1.44, *p* = 0.016)	1.41 (1.19–1.67, *p* < 0.001)	1.30 (1.10–1.55, *p* = 0.003)
>03:00	0.61 (0.40–0.94, *p* = 0.026)	0.82 (0.53–1.28, *p* = 0.393)	0.77 (0.49–1.20, *p* = 0.248)
Sleep time on weekends			
20:00–23:00	ref.	ref.	ref.
>23:00	1.04 (0.89–1.21, *p* = 0.658)	1.42 (1.20–1.67, *p* < 0.001)	1.34 (1.13–1.58, *p* < 0.001)
>03:00	1.11 (0.74–1.66, *p* = 0.610)	1.68 (1.10–2.55, *p* = 0.015)	1.51 (0.98–2.31, *p* = 0.059)

CI, confidence interval; OR, odds ratio; WCS, weekend catch-up sleep.

Model 1: there was no adjustment for covariates.

Model 2: race, age, and gender were adjusted.

Model 3: race, age, gender, education level, BMI, diabetes, hypertension, alcohol consumption, and smoking status were adjusted.

**Figure 2:**
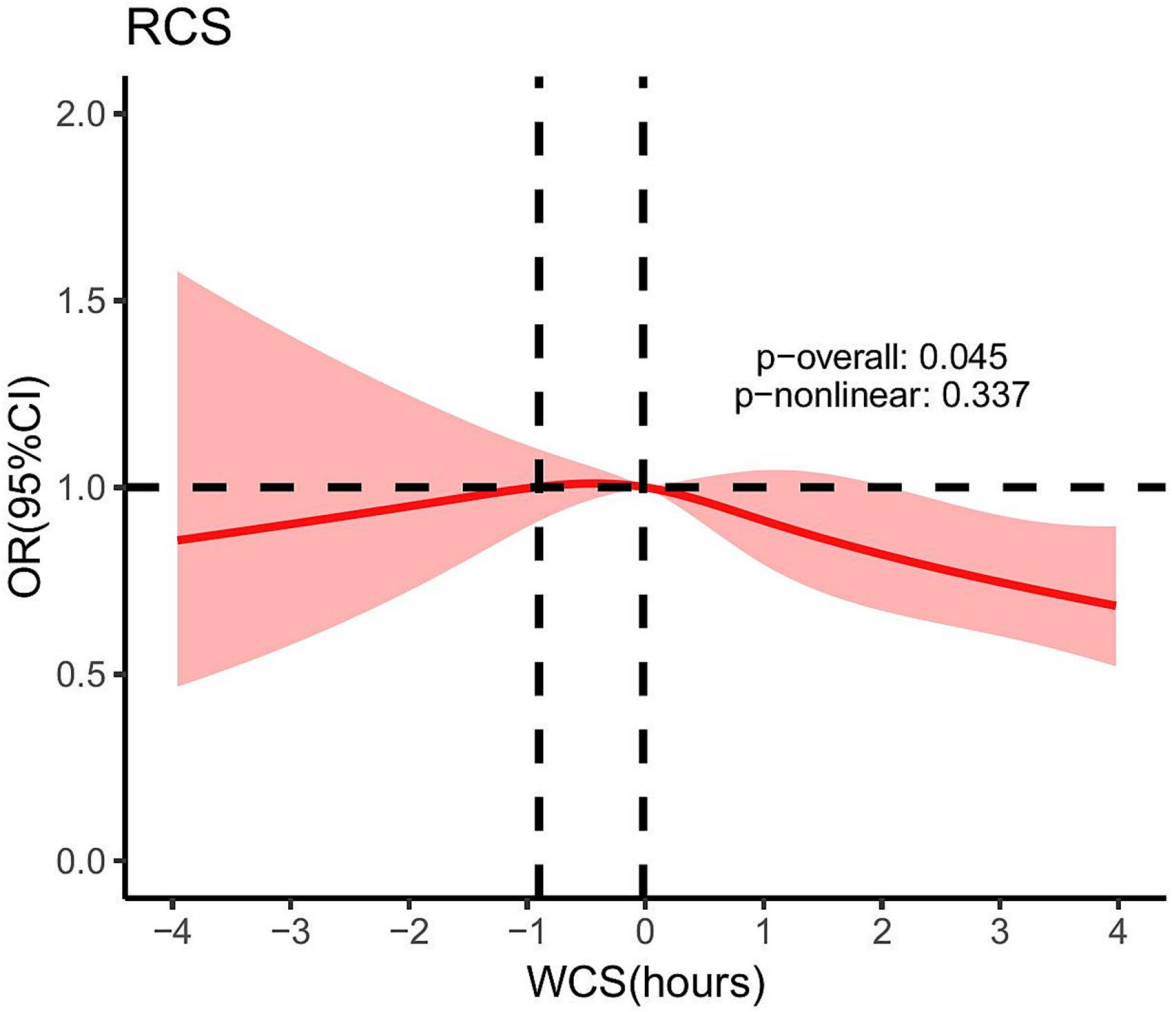
After adjusting for gender, race, education level, BMI, diabetes, alcohol consumption, and smoking status, the RCS for the association between WCS and gallstones. Dotted lines mean: At WCS values of −0.90 h and −0.02 h, the OR was equal to 1. WCS, weekend catch-up sleep; OR, odds ratio; RCS, restricted cubic spline.

As illustrated in Figure 2, after adjusting for covariates, namely gender, race, education level, BMI, diabetes, alcohol consumption, and smoking status, the RCS results showed that the *p*-value of the overall effect test (*p*-overall) was 0.045, indicating a significant association between WCS and gallstones. However, the *p*-value of the non-linear effect test (*p*-non-linear) was 0.337, suggesting that no significant non-linear relationship was found. Therefore, it can be concluded that the relationship between WCS and gallstones is linear.

### 3.3 Subgroup analyses

**Figure 3:**
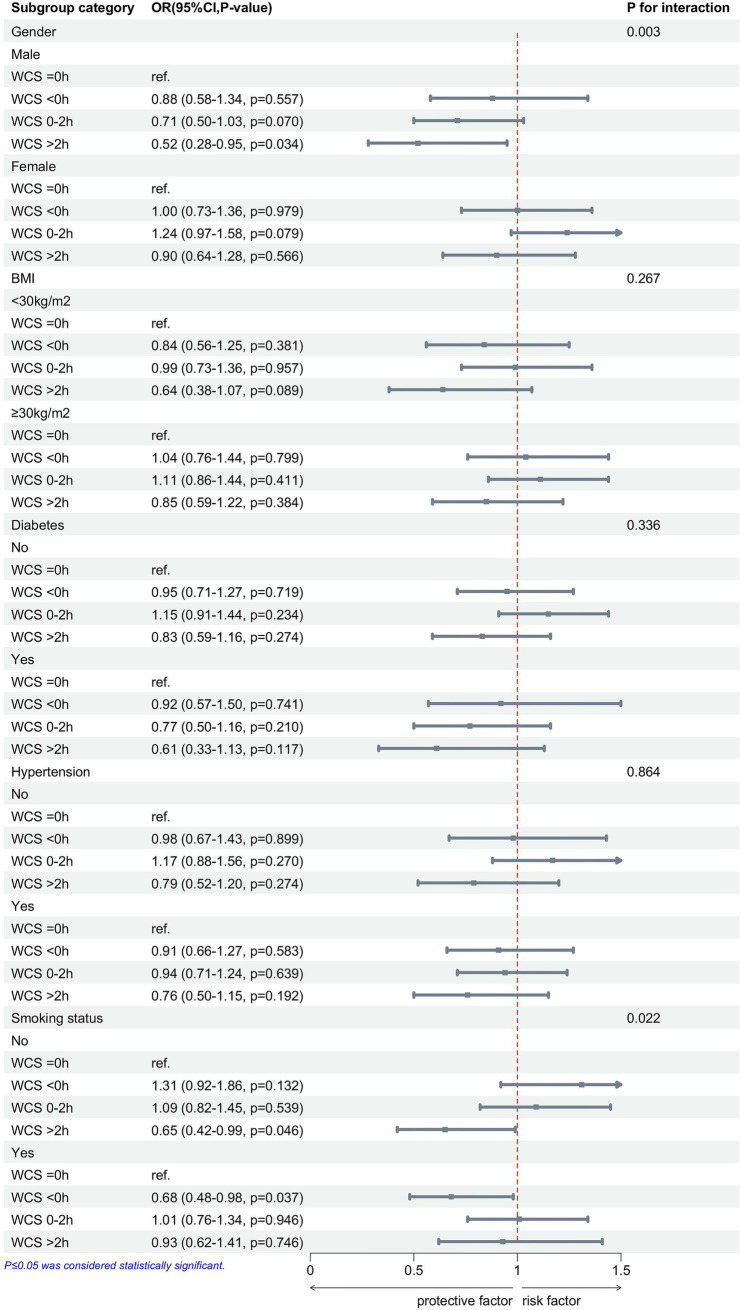
After adjusting for race, age, gender, BMI, education level, diabetes, hypertension, smoking status, and alcohol consumption, subgroup analyses show the associations between WCS and gallstones. WCS, weekend catch-up sleep; BMI, body mass index; OR, odds ratio; CI, confidence interval.

To further examine the relationship between WCS and gallstone disease, we categorized individuals based on gender, BMI, hypertension, diabetes, and smoking status, followed by a multivariable logistic regression analysis (Figure 3). There were significant interaction effects between gender (*p* for interaction = 0.003), smoking status (*p* for interaction = 0.022), and WCS on the risk of gallstones. In addition, we found that in the male, non-smoking participants, WCS > 2 h is a protective factor against gallstones, whereas in the smoking participants, WCS < 0 h served as a protective factor against gallstones.

## 4 Discussion

This cross-sectional study used NHANES data from 2017 to 2020 to examine the relationship between WCS, other sleep factors, and gallstones. The results of this study demonstrated that trouble sleeping, sleep time on weekdays and weekends, and WCS are closely connected with gallstones. In Model 1, unadjusted for any variables, both WCS < 0 h and WCS > 0 h were identified as protective factors against gallstones. In model 3, adjusted for all covariates, trouble sleeping and late sleeping were risk factors for gallstones. In addition, the RCS demonstrated a negative linear correlation between WCS and gallstones. In subgroup analyses adjusted for all covariates, WCS > 2 h was found to be a protective factor for gallstones in the male and non-smokers, but WCS < 0 h was a protective factor for gallstones in the smokers.

Gallstones are a major public health issue with high prevalence rates in the United States and Europe, where they are the most frequently encountered gastrointestinal disorders leading to hospital admissions (1, 26, 27). Sleep is a critical physiological process that modulates various metabolic and endocrine functions (9). Additionally, WCS has been linked to some health conditions (20, 22). In Table 1, we found that weekday sleep time was significantly associated with gallbladder stones, whereas weekend sleep time was not. One possibility is that weekday routines are more consistent, while weekend behaviors are influenced by diet (28). This may lead to a non-significant association between weekend sleep time and gallstones. In Table 2, we found that both WCS < 0 h and WCS > 0 h were protective factors for gallstones compared to WCS = 0 h. WCS < 0 h indicates that weekend sleep duration is shorter than weekday sleep duration, which typically corresponds to earlier waking times and earlier breakfast consumption on weekends. This may promote earlier bile secretion, preventing bile stasis and thereby reducing the risk of gallstone formation (29). It may also represent relatively longer daytime activity and increased exercise on weekends, thus reducing the incidence of gallstones (30). WCS > 0 h means weekend sleep duration is greater than weekday sleep duration. Previous studies have indicated that for each hour increase in WCS, BMI decreases by 0.12 kg/m^2^ (19), and WCS > 1 h can reduce the risk of developing hypertension (21). Obesity and hypertension are risk factors associated with an increased incidence of gallstones (6, 31), suggesting that WCS may reduce the prevalence of gallstones. In addition, the study by Han et al. found that WCS can reduce high-sensitivity C-reactive protein levels (23). C-reactive protein is an acute-phase protein secreted by the liver in response to infection, inflammation, or tissue injury, induced by pro-inflammatory cytokines (32). An inflammatory response in gallbladder epithelial cells may be triggered by elevated levels of circulating inflammatory proteins and cytokines, leading to reduced contractility, wall fibrosis, and epithelial damage (33–35). This dysmotility impairs gallbladder contraction and bile expulsion, creating conditions conducive to gallstone formation (36, 37). In the study by Jiang et al. (38), higher Log high-sensitivity C-reactive protein levels were associated with an increased risk of gallstones, particularly in younger individuals. This suggests that WCS may reduce gallstone formation by lowering high-sensitivity C-reactive protein levels. In our study, we also found that sleep time on weekends and weekdays is similarly connected with the presence of gallstones. All covariates were adjusted, and we found that late sleep and abnormal sleep time are correlated with a higher incidence of developing gallstones. This is consistent with the findings of Zhuang et al. (24), who reported that late sleep increases the prevalence of gallstones. One study demonstrated that circadian rhythm disruption leads to impairments in hepatic lipid metabolism and dysbiosis of gut microbiota in mice (39). Disruptions in lipid metabolism and gut microbiota are risk factors for gallstone formation, promoting the development of gallstones (1). Late sleep is often associated with prolonged exposure to light at night (40), as indoor light exposure before bedtime inhibits melatonin production and reduces its duration (41). Research indicates that melatonin can reduce gallstone formation by decreasing intestinal cholesterol absorption and enhancing gallbladder motility (42). In our study, we discovered that trouble sleeping is connected with a higher prevalence of gallstone disease, which aligns with the findings of Zhuang et al. (24). Trouble sleeping is associated with hypertension, obesity, and reduced vitamin C (43–45). These conditions, in turn, are linked to an increased risk of gallstone formation (6, 7, 31). Zhang et al. (24) identified short sleep duration as a risk factor for gallstones; however, our study found no significant correlation between either long or short sleep duration and gallstone incidence. We require large-scale prospective cohort studies to delineate the associations between them more comprehensively.

Subgroup analysis showed a significant association between WCS and gallstones, especially in the gender and smoking groups. Gender and smoking were strongly associated with gallstones, and male and non-smokers were protective factors for gallstones (5, 46). There is a close association between gender and sleep, with females exhibiting longer sleep duration but poorer sleep quality (47). This may cause WCS to be influenced by both sleep quality and sleep duration, resulting in gender differences in the protective effect of WCS against gallstones. Studies have shown a significant link between smoking and sleep, with smoking disrupting sleep structure, reducing sleep quality, and affecting sleep-related complications (48). WCS < 0 h usually implies an early breakfast, which may promote bile excretion and avoid cholestasis (29). It may also reflect differences in smokers’ lifestyle habits, and further biological and behavioral studies are needed to verify this. In addition, prospective studies and mechanistic experiments are needed to verify the interactive effects of gender, smoking, and WCS on gallstone formation.

Our research presents several notable advantages. First, the individuals in the NHANES study comprise an adequate representation sample of the U.S. population, meticulously adhering to a rigorously designed protocol and subjected to stringent quality control measures, thereby ensuring the validity of our findings. Second, we performed a comprehensive analysis of the relationships between WCS and other sleep-related factors with gallstone disease. Finally, through subgroup analyses, we further investigated the connection between WCS and gallstone disease among several demographic categories.

Nonetheless, our research also has inherent restrictions. First, because it is a cross-sectional study, the causality between WCS and gallstones remains to be clarified. Second, the NHANES data are derived from self-reported questionnaires, which may introduce recall bias. Despite these limitations, this study offers novel perspectives on the relationship between WCS and the prevalence of gallstone disease.

In conclusion, our research indicates that WCS can lower the incidence of gallstones, especially when WCS > 2 h, while trouble sleeping and late sleep can increase this incidence. In addition, the protective effect of WCS > 2 h on reducing the incidence of gallstones is particularly significant in individuals who are non-smokers and males, whereas in the smoking population, WCS < 0 h serves as a protective factor against gallstones.

## Data Availability

Publicly available datasets were analyzed in this study. This data can be found: https://www.cdc.gov/nchs/nhanes/.
